# Chronic Disease Mapping, an Important Strategy and Tool for Health Promotion

**DOI:** 10.5888/pcd21.240110

**Published:** 2024-04-25

**Authors:** Karen Hacker, Rachel Kaufmann

**Affiliations:** 1 National Center for Chronic Disease Prevention and Health Promotion, Centers for Disease Control and Prevention, Atlanta, Georgia

Loss of life from the COVID-19 pandemic has been tremendous over the past several years (1); however, chronic diseases like heart disease and cancer still account for the largest numbers of deaths in the US. Stroke and Alzheimer disease are also among the leading causes of death (2). Chronic disease overall continues to drive national mortality and morbidity (2). Its annual national medical cost exceeds $1 trillion, which doesn’t include the cost to the economy of workdays lost to illness and disability (3). Having a chronic disease like diabetes or cancer is a risk factor for severe morbidity and mortality from COVID-19 (4). We know that many chronic diseases can be prevented (5) and that risk behaviors such as tobacco use, alcohol consumption, poor nutrition, and lack of physical activity are the leading contributors to preventable chronic disease (6). As we move forward, our country’s ability to remain resilient is dependent on chronic disease prevention and management (7).

The Centers for Disease Control and Prevention’s (CDC’s) National Center for Chronic Disease Prevention and Health Promotion (NCCDPHP) is dedicated to preventing chronic disease and promoting health and wellness for all. Our 9 divisions work in major areas related to both risk factors such as smoking and physical inactivity and diseases such as cancer, diabetes, and cardiovascular disorders (7). Strategies fall into 4 domains: epidemiology and surveillance to understand the prevalence and incidence of conditions and behaviors over time, environmental approaches aimed at shifting behaviors and offering opportunities for healthy living to all, health care interventions that identify disease early and help manage chronic conditions, and connecting people to the clinical care and resources they need to thrive (8). NCCDPHP relies on various types of public health surveillance data, such as individual interviews about health behaviors, clinical and laboratory data, tracking cancer survivors’ medical journeys, sales data documenting Americans’ use of tobacco and food, and vital statistics from birth and death certificates (9). These are used to understand the population’s health status and trends, identify emerging issues, and evaluate whether interventions aimed at improving health have been successful.

Helping communities, state and local partners, and all interested parties understand the prevalence of chronic disease is always a challenge. Over the last few decades, visualization has become enormously helpful (10). Mapping information that helps people literally see where conditions disproportionately affect specific areas and groups has proved enlightening. Maps created by using geographic information systems — GIS — provide the public with clear, easy-to-understand information on patterns, relationships, and levels of disease or behavior within specific geographic areas (11). For example, a study of life expectancy at birth showed disparities as large as 20.1 years across US counties, with the lowest life expectancies clustered in the Southeast and Appalachia and the highest clustered in Colorado and the California coast (12).

Animated maps can also depict changes over time. Take for example maps of the obesity epidemic, which provided a stark understanding of the epidemic’s expansion (13). Or maps of opioid overdoses, which demonstrated the severe loss of life that occurred from 2011 through 2017 (14). More recently, maps of COVID-19 morbidity linked with maps of chronic disease helped local communities direct vaccination and other mitigation efforts (15). The value of the “Aha!” moment that occurs when you see the public health surveillance data in a GIS visualization cannot be overestimated. For example, in 1995, a map of blood lead testing results for young children attending WIC (Special Supplemental Nutrition Program for Women, Infants, and Children) clinics in Salt Lake County, Utah, showed that 76% of children with elevated blood lead levels resided in a contiguous area comprising 10% of the county (16). Consequently, the Salt Lake city and county health departments reached out to parents and physicians to encourage screening of young children living in that area. Screenings increased significantly, and additional children with elevated levels were identified. This early mapping application was uncomplicated yet revealing, providing the exact information local health departments needed to take appropriate action.

Although mapping for public health action may have begun with John Snow’s famous demonstration in 1854 of a cholera-contaminated water source (17), use of GIS in public health has proliferated over the past several decades. Today’s digital maps can involve multiple layers integrating disparate types and sources of information. GIS allows users to create maps that can examine health-related factors by location, elevation, and time. Users can integrate relevant information about population density, air quality, neighborhood wealth index, transportation routes, and food availability, as just a few examples. These “geospatial determinants of health” (18) need to be identified and shared with the people who set policy, plan interventions, treat patients, and organize communities.

In recent years, NCCDPHP has used GIS extensively to identify areas of high and low disease prevalence, and environments that dispose populations to high and low risk of chronic disease. These locations might benefit from directed interventions, producing changes over time. NCCDPHP has also sponsored efforts to increase the use of GIS by health departments. From 2018 through 2020, NCCDPHP’s Division of Heart Disease and Stroke Prevention published *GIS Express for Chronic Disease*, a newsletter for public health professionals to share GIS-related information (19). NCCDPHP has also supported the National Association of Chronic Disease Directors’ GIS Capacity Building Project, which provides GIS training for state and local health departments and established the Chronic Disease GIS Network to connect, support, and highlight public health professionals using GIS to address chronic disease priorities (20,21).

This *Preventing Chronic Disease* collection features 6 peer-reviewed articles that highlight examples of NCCDPHP’s uses of GIS in preventing and addressing chronic diseases. Most were submitted in 2023 in response to a call for papers in the journal’s article category, “GIS Snapshot,” and one essay featuring GIS maps was published before the journal’s call for papers. GIS Snapshots are intended to highlight the public health application of maps in a brief format, demonstrate how GIS informs chronic disease prevention and treatment, and inspire others to use GIS in their work (22). The articles in this collection document how GIS can be used to identify populations at greatest risk, locations for public health interventions, and sometimes-surprising relationships requiring more in-depth research.

The essay by Petersen et al (23) includes maps illustrating how obesity prevalence varies startlingly across the United States — not just by region, but also by race and ethnicity. While the obesity epidemic has affected the entire nation, its burden falls especially on non-Hispanic Black and Hispanic Americans. The Evans et al article (24) also examined national disparities by race and by county, this time for stroke. They found that counties with the highest number of stroke deaths were similar for Black and White Americans, but counties with the highest stroke hospitalization rates had more divergence, a finding that suggests avenues for future study in stroke care. 

Geolocating areas where resources are needed can be useful for decision makers as they consider interventions directed at patients, clinicians, and the general public. In the Wittman et al (25), Fujii et al (26), and Richardson et al (27) articles, authors sought information critical for focusing these future interventions. Wittman et al found that in Appalachia, economically distressed counties are less likely to have a diabetes self-management program, even though having to travel a long distance to participate may be an important barrier to program use in these communities. Such analyses can provide decision makers with important information about where new programs are needed to improve access. In a similar vein, Fujii et al examined locations of barber and beauty shops as potential community-based resources in fighting hypertension. Their analysis demonstrates the potential feasibility of bringing the LA Barbershop Model (28), in which blood pressure screenings are offered at community-friendly locations, to other cities. Richardson et al examined state-level improvements in colorectal cancer screening rates to elucidate patterns of use and opportunities for improvement. Although screening prevalence has increased in every state since 2012, 22 states did not meet the national target screening rate for 2020. Lastly, GIS visualizations can also prompt additional unanswered questions. For example, the analysis by Han et al (29) of chronic kidney disease and poverty at the county level showed that outcomes do not always follow predicted patterns. Poverty and chronic kidney disease were not always related as expected, and the pattern seemed to vary by region.

The articles in this collection demonstrate just a few recent uses of GIS at NCCDPHP. Mapping is used extensively by CDC programs and partners to highlight features such as prevalence and geographic distribution of risk factors, disease outcomes, and community characteristics. Geographic visualizations can be important tools during emergency responses but also play a key role in understanding relationships among disorders, risk factors, environmental context, and other factors. In 2019, *Preventing Chronic Disease* published an article collection, *Population Health, Place, and Space: Spatial Perspectives in Chronic Disease Research and Practice.* The articles in that collection provided insights on how using GIS mapping advances understanding of connections between community-level characteristics and population health and showed innovative ways of developing and applying new spatial statistical methods and geospatial tools in public health and how maps and geospatial results can be used to guide program and policy decisions (30). 

Today, GIS competency is necessary for public health departments across the nation at local, county, and state levels (31). Its use will continue to evolve, and we look forward to applying it to additional chronic disease issues. As artificial intelligence becomes more available, this too will help to drive GIS capacity, such that large datasets can be transformed into clearly visible spatial analyses (32). For further information on the work across NCCDPHP and to download state and local chronic disease data for your own GIS maps, visit the National Center for Chronic Disease Prevention and Health Promotion (NCCDPHP), Open Data Portal (www.cdc.gov/chronicdisease/data/indicators.htm).
